# Disease Termed “Black Leg,” among the Ottawa Lumbermen

**Published:** 1864-02

**Authors:** 


### DISEASE TERMED "BLACK LEG," AMONG THE OTTAWA LUMBERMEN.

Dr. J. O. Grant describes (*Med. Times and Gaz.*, Dec. 26, 1863) a disease of this name prevalent among Ottawa lumbermen.

"In one shanty," he states, "twenty-five men out of thirty-six were attacked with this disease, and, from ascertained facts, the great proportion of the cases were developed as follows:—

Slight pains in the extremities, particularly about the ankle joints and posterior parts of the legs. After a few days, in severe cases, the pain is liable to extend to the arms and shoulder joints. The integument of the legs is first observed to change color, passing from a somewhat yellow to a deep venous hue, in large patches, almost approaching to a black (hence the term.) The legs and the arms are liable to swell, particularly the former. Frequently, two or three weeks before any constant pain is complained of, or change of color takes place, the limbs move sluggishly in response to the will, and considerable soreness is experienced upon pressure. Abrasion of the integument is followed by a sero-sanguinolent discharge; and, if much irritated, is liable to inflammation, partaking of the asthenic character. The limbs are said to be almost free from pain when immersed in water during the spring season rafting; but afterwards they become hard, painful, and stiff. The gums are frequently observed swollen and spongy for some weeks before the limbs become painful. Bowels usually regular, and urine voided in normal quantity. Sleep restless. Many of the men were subject to headache, giddiness, loss of appetite, and swelling of the eyelids; also, at times, to a peculiar sensation, as if the head had attained enormous dimensions. During the month of April the great proportion of these cases became most marked, and, under judicious treatment, rarely extended over a period of four weeks before convalescence was established. It was not an unfrequent circumstance to observe, amongst those who were exposed to the same dietary influence, attacks of acute rheumatism, as well as nyctalopia, both of which readily yielded to rest and regimen, in conjunction with mild medicinal agents. Whenever nyctalopia is detected by the experienced lumberer, fresh milk is administered largely, when obtainable, which has a most speedy and salutary influence, the retina recovering its tone in the space of a few days.

"This disease, from its particulars, appears to class with scorbutus, being from all appearances an aggravated variety, resulting not alone from a sameness of diet, but also from the influence of nitrate of potash upon the blood. This salt is largely used by the packers to preserve the pork in the summer season. During the early lumbering operations, twenty-five or thirty years ago, on the rivers Ottawa and Gatineau, the occurrence of this disease was very frequent, owing in a great measure to the extensive use of this salt of potash. The trade and experienced packers, being aware of these facts, now have recourse to this material only in moderation, an excess not being necessary to prevent putrefaction taking place, in consequence of which this disease is now seldom observed. Dr. Garrold states (*Monthly Journal of Medical Science*, January, 1848), that from an examination of the composition of the food, etc., etc., he was led to the conclusion that the absence of potash was the cause of scurvy. Notwithstanding the accuracy of these observations, it is a fact well tested through a process of years, that when any excess of nitrate of potash is used to preserve the staple article of diet, pork, this scorbutic black leg is liable to be developed."

